# e-Counseling for Fall Prevention in Older Adults: Scoping Review

**DOI:** 10.2196/78444

**Published:** 2026-05-26

**Authors:** Lina Safarina, Cecep Eli Kosasih, Iqbal Pramukti, Nucki Nursjamsi

**Affiliations:** 1 Department of Nursing, Faculty of Nursing Universitas Achmad Yani Cimahi, West Java Indonesia; 2 Department of Nursing, Faculty of Nursing Universitas Padjadjaran Sumedang Indonesia

**Keywords:** e-counseling, fall prevention, older adults, digital health, telehealth, scoping review

## Abstract

**Background:**

Falls are a major contributor to injury, disability, and mortality among older adults worldwide. Digital health–based e-counseling has emerged as a scalable strategy to support fall prevention by addressing physical, behavioral, and cognitive risk factors.

**Objective:**

This scoping review aimed to map the scope, characteristics, and outcomes of e-counseling interventions for fall prevention among adults aged 60 years or older.

**Methods:**

Guided by Arksey and O’Malley’s framework and reported in accordance with PRISMA-ScR (Preferred Reporting Items for Systematic Reviews and Meta-Analyses Extension for Scoping Reviews), we searched PubMed, ScienceDirect, CINAHL, and ProQuest for studies published between January 2019 and May 2025. Eligible studies evaluated digital or telehealth-based counseling interventions for fall prevention. Data were synthesized descriptively, and methodological quality was appraised using the Mixed Methods Appraisal Tool.

**Results:**

A total of 13 studies were included, comprising randomized controlled trials, feasibility studies, qualitative research, and retrospective cohorts conducted across Asia, Europe, and North America. e-Counseling modalities included virtual reality, mobile health application, teleconsultations, and wearable-triggered feedback systems. Reported outcomes included reductions in fall incidence ranging from 15% to 90.9%, improvements in balance and mobility, increased fall risk awareness, and enhanced self-efficacy. Interventions incorporating cultural tailoring and digital literacy support demonstrated higher engagement and adherence.

**Conclusions:**

e-Counseling appears to be a promising and scalable approach for fall prevention in older adults, with benefits across physical and behavioral domains. Future research should focus on long-term effectiveness, equitable access in low- and middle-income settings, and integration into routine health care systems.

## Introduction

The rapid aging of the global population is transforming societal structures and health care demands worldwide. The global population aged 60 years and older is expected to double, reaching 2.1 billion by 2050, with 16% of the world’s population being 60 years or older by 2050 [1]. In Indonesia, 26 million people are aged 60 years and older, accounting for 9.8% of the total population [2]. The population of older adults is expected to reach 48 million by 2045, accounting for 15% of the total population [3]. This significant growth in the older adult population underscores the increasing demographic importance of this age group and the urgent need for attention to their needs and health challenges.

Falls are one of the leading causes of morbidity and mortality among older adults, often resulting in severe physical injuries, psychological distress, and increased health care costs [4]. Globally, it is estimated that 646,000 older adults die from falls each year, making it the second leading cause of accidental deaths worldwide [4]. In the United States, about 36 million falls are reported among older adults each year, leading to more than 32,000 fatalities [5]. In Indonesia, falls are also a major health issue, with approximately 23% of older adults experiencing at least 1 fall annually, contributing to significant morbidity and health care costs [6,7]. Contributing factors to the increased risk of falls include age-related physical decline, such as decreased muscle strength and balance, as well as chronic health conditions such as arthritis, visual impairments, and cognitive decline [8,9]. Furthermore, polypharmacy, or the use of multiple medications, can increase the likelihood of dizziness and instability, further elevating the risk of falls [10,11]. These alarming statistics underscore the urgent need for targeted interventions to prevent falls and promote the overall health and safety of the older adult population.

Falls among older adults have significant physical, emotional, and economic consequences, making them a critical public health concern. Physically, falls are the leading cause of injury and injury-related mortality in this demographic, accounting for approximately 646,000 deaths globally each year [4]. Survivors often experience serious injuries, including fractures and head trauma experience, leading to long-term disabilities and increased dependence on caregivers [12,13]. Emotionally, many older adults develop a fear of falling again, which can result in reduced physical activity, social isolation, and a diminished quality of life [14]. Economically, falls impose a substantial burden on health care systems, with US medical costs for fall-related injuries exceeding US $50 billion annually [5]. This financial strain affects not only individuals but also families and health care resources, highlighting the urgent need for effective fall prevention strategies to mitigate these impacts [15,16].

Various preventive measures have been implemented to address this critical issue. Traditional strategies, such as balance and strength training interventions, have shown effectiveness in reducing fall risk by improving physical capabilities [17]. Home safety assessments and modifications, including removing tripping hazards and improving lighting, are also essential strategies [18]. Additionally, community-based interventions that combine education and physical activity, such as Tai Chi classes, have been proven to enhance balance and reduce falls among older adults [19]. However, these interventions are not always accessible, particularly for older adults with mobility limitations or those living in rural and underserved areas [18]. In response, digital health innovations, particularly e-counseling, have emerged as promising tools for delivering tailored fall prevention strategies in a more scalable and accessible format.

In this context, e-counseling refers to digitally delivered, personalized counseling provided via telehealth platforms, mobile applications, or online systems to support behavior change e-counseling refers to digitally delivered, personalized counseling provided via telehealth platforms, mobile application, or online systems to support health behavior change. In fall prevention, e-counseling facilitates individualized goal setting, progress monitoring, and behavioral coaching grounded in self-efficacy theory [20]. Evidence suggests that e-counseling can improve fall risk awareness, adherence to preventive behaviors, and engagement in balance and mobility exercises [21,22]. By overcoming barriers related to mobility, distance, and resource constraints, e-counseling offers a scalable alternative to traditional in-person interventions. However, evidence remains fragmented regarding long-term sustainability, effectiveness across diverse populations, and integration into existing health care systems, highlighting the need for a comprehensive scoping review [23-26].

Despite its growing application, the evidence base on e-counseling for fall prevention in older adults remains fragmented [27]. Prior reviews have largely focused on in-person interventions or general eHealth approaches, with limited synthesis specifically addressing e-counseling modalities and outcomes. Additionally, there is a paucity of evidence on how these interventions are tailored for diverse populations, integrated into clinical practice, and sustained over time. Given these gaps, a scoping review is warranted to map the scope, characteristics, and outcomes of existing e-counseling interventions aimed at fall prevention among older adults. Therefore, this scoping review aims to (1) map the types and delivery modes of e-counseling interventions for fall prevention; (2) summarize reported physical, cognitive, and behavioral outcomes; and (3) identify research gaps to inform future intervention development and implementation.

## Methods

### Study Design

This study employed the methodological framework established by Arksey and O’Malley for conducting a scoping review [28] used, which consisted of 5 essential steps. First, the research question was clearly and objectively identified. Second, relevant articles were selected to ensure the study’s focus. Third, related literature was extracted from the identified articles for further analysis. Fourth, the data collected from these sources were organized, summarized, and analyzed. Finally, the findings were reported, as outlined in the framework [28]. The review also adhered to the PRISMA-ScR (Preferred Reporting Items for Systematic Reviews and Meta-Analyses Extension for Scoping Reviews) checklist to ensure comprehensive and replicable reporting [29].

### Research Question and Framework

The review was guided by the population, concept, and context framework: population—older adults aged ≥60 years at risk of falls; concept—e-counseling interventions for fall prevention, including digital, telehealth, or app-based personalized counseling; and context—health care or community-based settings where digital interventions are implemented. The central research question was as follows: *What is the current scope of evidence regarding the use of e-counseling to prevent falls in older adults?*

### Eligibility Criteria

Studies were included if they met the following criteria: (1) population—participants aged 60 years or older with a focus on fall prevention; (2) intervention—e-counseling interventions delivered through telehealth platforms, mobile application, video consultations, or other digital formats; (3) outcomes—reports on fall rates, balance, mobility, adherence, or related physical and cognitive outcomes; (4) study types—peer-reviewed empirical studies (quantitative, qualitative, or mixed methods), including randomized controlled trials (RCTs), quasi-experimental studies, longitudinal studies, and cohort studies; (5) timeframe—published between January 1, 2019, and May 31, 2025; and (6) language—English. Studies were excluded if they focused solely on in-person counseling, targeted populations other than older adults, or lacked full-text availability.

### Information Sources and Search Strategy

Literature searches for this study were conducted using PubMed, ScienceDirect, CINAHL, and ProQuest in May 2025. The search strategy was designed to provide a comprehensive overview of e-counseling interventions for fall prevention among older adults. Keywords were aligned with Medical Subject Headings (MeSH) and incorporated Boolean operators “OR” and “AND” to refine the search results. The specific keywords used included “e-counseling,” “fall prevention,” “older adults” OR “elderly,” “telehealth” OR “digital counseling.” This systematic approach ensured that relevant literature was thoroughly explored to inform the study’s objectives.

### Identification and Selection of Literature

The literature search was conducted independently through trusted databases. The retrieved articles were then analyzed, with differences and duplicates being removed. The process of searching for and selecting articles was illustrated using the PRISMA-ScR flowchart [30].

### Data Extraction

Data were collected and organized in Microsoft Word in the form of a table containing several components: article title, authors, publication year, research objectives, study design, study location, type and delivery mode of e-counseling intervention, technologies or platforms used, fall-related outcomes (eg, fall rates, balance, and mobility), cognitive or behavioral outcomes (eg, adherence and self-efficacy), and key findings. Two reviewers performed data extraction independently to ensure consistency and accuracy.

### Quality Appraisal

While scoping reviews do not typically require formal quality evaluation, this review incorporated a structured assessment to better understand the methodological soundness of the included studies. To achieve this, each study was appraised using the 2018 version of the Mixed Methods Appraisal Tool (MMAT), with consideration given to its specific research design. Two independent reviewers conducted the appraisal, examining elements such as the clarity of the research aim, the suitability of the chosen methods, the completeness of reported outcomes, and potential sources of bias. Importantly, this evaluation was not used to determine study inclusion or exclusion but served to enhance the interpretation of findings and highlight methodological gaps that could guide future investigations.

### Data Synthesis and Analysis

A descriptive and thematic synthesis approach was applied. Data were first categorized based on study design, intervention type, and outcome domains. Themes were then identified around the delivery formats (eg, telehealth and mobile apps), effectiveness (eg, fall reduction and mobility improvement), and implementation factors (eg, adherence and accessibility). Findings were summarized narratively, supported by tabulated evidence to illustrate patterns, strengths, and gaps.

## Results

### Search Results

**Figure 1:**
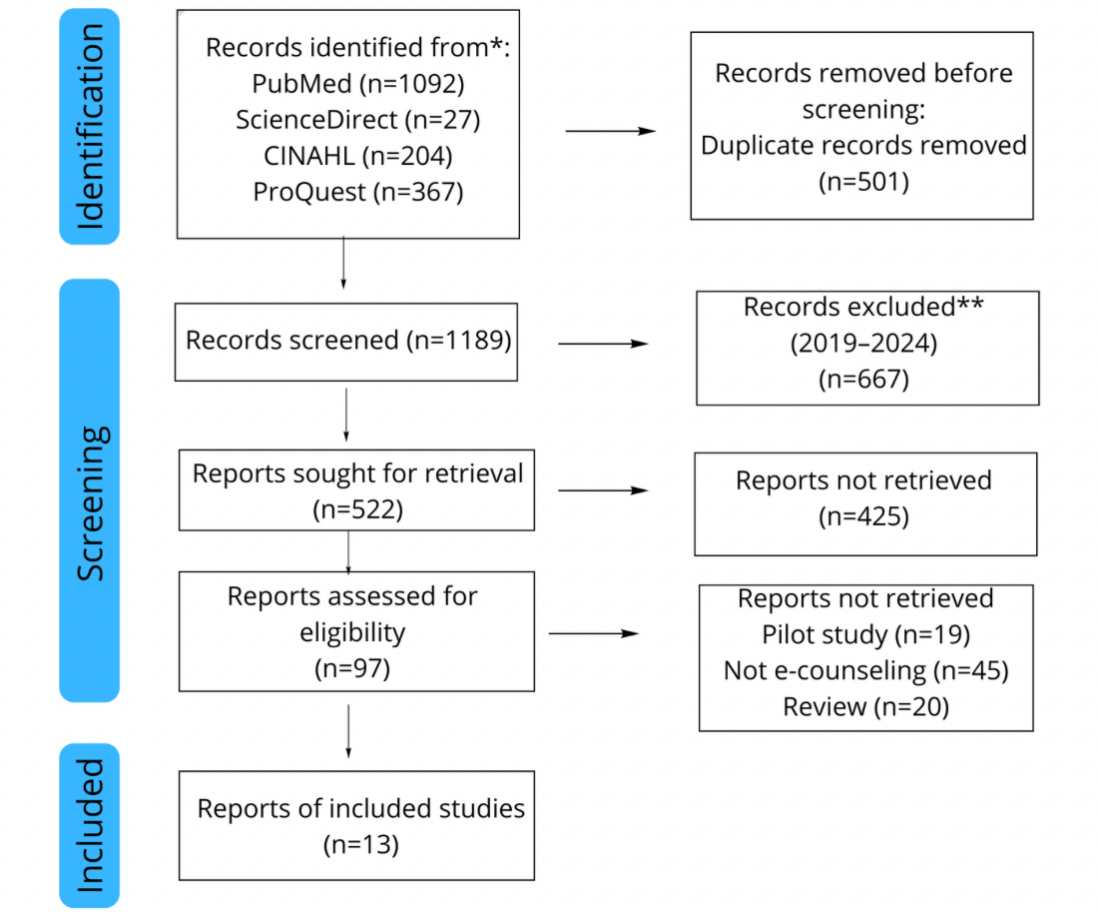
PRISMA-ScR (Preferred Reporting Items for Systematic Reviews and Meta-Analyses Extension for Scoping Reviews) flow diagram illustrating the study selection process for this scoping review. n indicates the number of records at each stage, and N indicates the total number of records identified. The figure is adapted from Moher et al [31]).

The flow of study selection is outlined through the PRISMA-ScR flow diagram, detailing each phase of the review process. An initial search across PubMed, ScienceDirect, CINAHL, and ProQuest yielded 1690 records. After eliminating 501 (29.6%) duplicates, 1189 (70.4%) titles and abstracts were screened. The remaining 97 articles underwent full-text review. During this phase, 84 studies (86.6%) were excluded: 19 (22.6%) were pilot trials, 45 (53.6%) did not involve e-counseling, and 20 (23.8%) were review papers. In the end, 13 studies (13.4%) were included in the final analysis. The study selection process is presented in the PRISMA-ScR flowchart (Figure 1; [30,31]).

### Findings

#### Characteristics of Included Studies

The included studies demonstrated a diverse range of research designs. RCTs [32,33] offered strong empirical evidence on intervention effectiveness. Pilot and feasibility studies [34,35] assessed initial usability and participant acceptance. Qualitative designs [36,37] provided insight into user perceptions, while retrospective cohort analyses [38,39] and observational designs [40] reflected real-world outcomes. Thiamwong et al [41] applied a cluster RCT to evaluate community-based peer-led interventions. The geographical distribution of the studies reflects global interest in digital fall prevention. Studies were conducted in Asia (Hong Kong, Thailand, and Indonesia), Europe (Germany and the United Kingdom), and North America (the United States). This variety shows the adaptability of e-counseling across different health care systems and cultural contexts.

Delivery modes ranged from immersive virtual reality (VR) [32], app-based learning modules [42], and artificial intelligence (AI)–assisted mobile health (mHealth) apps [39] to telehealth-facilitated interventions [33,43]. Peer-supported and community-led interventions [41] and hybrid models that transitioned from in-person to digital [34] highlight the flexibility of intervention formats. Wearable-triggered interventions, such as sensor belts [44], were also represented. The platforms used included tablet application [40], full-immersion VR systems [32], wearable smart belts [44], and wrist-worn fitness trackers [36]. mHealth application [39] and telehealth delivery models [43] were common. Digital literacy training [34] was crucial in improving accessibility among older adults.

#### Quality Appraisal Results

The pilot RCTs by Ip et al [32] and Arena et al [33] met most MMAT criteria, demonstrating well-defined aims, suitable randomization methods, and consistent outcome measures. Both studies had clear intervention protocols and provided sufficient follow-up data, although sample sizes were relatively small, limiting generalizability.

Dajpratham et al [38] and Alves et al [39], using retrospective designs, demonstrated strong documentation of real-world outcomes and substantial sample sizes. However, their reliance on previously collected data posed risks of selection and reporting biases, particularly in the absence of control groups or standardized assessment timelines. Community-based interventions such as those conducted by Nazhira et al [42] and Thiamwong et al [41] provided robust insights into localized fall prevention strategies. While their designs were appropriate for evaluating feasibility and community relevance, some limitations were noted in participant blinding and long-term outcome measurement.

Feasibility and observational studies by Hladek et al [34], Jacobson et al [35], and Bajdek et al [40] generally adhered to MMAT standards, showing clearly defined objectives and consistent methods. However, several studies lacked comprehensive reporting on confounding variables and did not always include detailed rationales for sample size selection.

Qualitative studies, including those by Ojo et al [36] and Tang et al [37], scored highly on relevance and depth of data interpretation, effectively capturing user experiences and motivational factors. Nevertheless, transparency regarding coding frameworks and researcher reflexivity was limited in some cases.

The intervention led by Ritchey [43], though pilot in scope, aligned with MMAT criteria for interventional research, showcasing clear protocols and outcome metrics. Similarly, the real-world adaptive study by Kinosian and Stefanacci [44] demonstrated a high degree of ecological validity and intervention relevance, though it lacked some methodological transparency in data analysis.

#### Key Findings

Across the included studies, several consistent patterns emerged. First, e-counseling interventions were delivered through diverse digital modalities, including VR, mobile application, telehealth sessions, and wearable-based feedback systems [32]. Despite this variation, effectiveness did not appear to depend solely on technology type. Instead, interventions that incorporated personalized feedback, cultural tailoring, and usability support demonstrated higher engagement and adherence [43]. Second, most studies reported improvements in balance, mobility, and fall-related outcomes, with reported reductions in fall incidence ranging from 15% to 90.9% [39]. Third, behavioral and psychological outcomes—such as increased self-efficacy, confidence, and fall risk awareness—were commonly reported, particularly in interventions that emphasized goal tracking and continuous monitoring. Barriers to implementation included limited digital literacy, unequal access to technology, and resource constraints, especially in underserved populations [44].

Positive effects of these interventions were noted across a range of outcomes, particularly those related to fall risk. Improvements were frequently reported in participants’ mobility, balance, and overall fall incidence. For example, the use of VR led to significant gains in both physical coordination and cognitive functioning [32]. Similarly, tools that used AI to assess fall risk enabled more accurate predictions and preventive action [39]. Interventions delivered through peer coaching and app-based platforms demonstrated measurable reductions in fall risk and improvements in physical capability [33,41]. Notably, the remarkable 90.9% reduction in hip fractures reported with the Tango Belt (ActiveProtective Technologies, Inc) highlighted the value of wearable, sensor-based protective devices.

Beyond physical outcomes, psychological and behavioral effects were also evident. Several studies recorded notable increases in participants’ confidence in managing fall risk, better executive functioning, and improved knowledge of fall prevention techniques. Interventions that focused on building digital skills and promoting access, especially among vulnerable groups, also led to increased confidence and social connectedness [34]. Key to behavior change was the use of individualized feedback and goal tracking features, which fostered greater involvement and sustained commitment from users. The success of these interventions was also closely tied to how well they were implemented. User satisfaction and acceptance were higher in studies that emphasized intuitive design and meaningful interaction. At the same time, disparities in digital access, technological literacy, and economic resources emerged as ongoing barriers to equal adoption. These challenges point to the need for thoughtful planning when considering how such interventions might be scaled and integrated more broadly. The characteristics of the included studies are summarized in Table 1.

**Table 1:** Characteristics of the studies and summary of the findings (N=13).

Authors and publication year	Study design	Study location	Type and delivery mode of e-counseling	Technologies or platforms used	Fall-related outcomes	Cognitive or behavioral outcomes	Key findings
Ip et al [32], 2025	Pilot RCT^a^	Community setting, Hong Kong	VR^b^ game-based interactive training	Full-immersive VR system	Mobility, balance, walk speed	Executive function, fall efficacy	Greater cognitive-motor improvement vs non-VR group
Dajpratham et al [38], 2025	Retrospective cohort study	Thailand	Personalized digital fall risk assessment	Clinical + digital tools	Fall rate, balance (Timed Up and Go, 30-s chair stand)	Medication adherence	Falls reduced from 80% to 27.4%; sedatives increased risk
Nazhira et al [42], 2025	Community intervention	Indonesia	Video-based balance training	Digital instructional videos	Balance improvement	Knowledge gain	Improved balance and daily confidence
Alves et al [39], 2024	Retrospective real-world analysis	Germany	AI^c^-assisted risk feedback	Longitudinal INdividualized Digital Evaluation of Risk and Aging mHealth^d^ app	Fall incidence (6–24 months)	Risk awareness, engagement	Fall Risk Score predicts falls; supports stratified care
Bajdek et al [40], 2024	Observational study	United States	Digital exercise + cognitive assessment	Tablet-based app	Exercise adherence	Cognition, tech readiness	High adherence improves cognition
Ritchey et al [43], 2024	Interventional Veterans Affairs pilot)	United States	Virtual exercise + education	Telehealth platform	Balance, sit-to-stand	Fall self-efficacy	High feasibility and independence
Kinosian et al [44], 2024	Adaptive cohort	United States	Wearable protection system	Tango Belt	90.9% reduction in hip fractures	Adherence	Effective injury prevention
Ojo et al [36], 2024	Qualitative PEER^e^ follow-up)	United States	Wearable engagement intervention	Fitbit	Increased physical activity	Motivation	Real-time feedback improves adherence
Tang et al [37], 2024	Qualitative study	United Kingdom	Digital instructional app	Keep On Keep Up (Chinese)	Balance awareness	Engagement, usability	Cultural tailoring improves uptake
Thiamwong et al [41], 2024	Cluster Randomized Controlled Trial	United States	Peer-led digital coaching	BTrackS	Reduced fall risk behavior	Perception, adherence	Effective in low-income groups
Hladek et al [34], 2023	Feasibility study	United States	Hybrid digital transition	Digital literacy + device training	Not directly measured	Confidence, inclusion	Improves access and digital skills
Arena et al [33], 2021	Randomized Controlled Trial	United States	HOP-UP-PT program^f^	In-person + phone	Reduced fall risk	Mobility confidence	Effective home-based intervention
Jacobson et al [35], 2021	Feasibility study	United States	Digital coaching	Online modules	Feasibility outcomes	Usability, acceptance	Scalable and acceptable

^a^RCT: randomized controlled trial.

^b^VR: virtual reality.

^c^AI: artificial intelligence.

^d^mHealth: mobile health.

^e^PEER: Participant Engagement and Empowerment Resources

^f^HOP-UP-PT program: Home-based Older Persons Upstreaming Prevention–Physical Therapy.

## Discussion

### Principal Findings

This scoping review demonstrates that e-counseling interventions offer meaningful benefits for fall prevention among older adults, with consistent improvements observed in physical outcomes, behavioral engagement, and fall risk awareness across diverse settings.

The study findings demonstrate that e-counseling offers substantial potential in addressing the multifaceted issue of fall prevention among older adults. The diverse modalities used, ranging from VR training to mHealth application and wearable technologies, demonstrate adaptability to various settings and populations. Notably, the integration of culturally tailored content and user-friendly interfaces appears to enhance engagement and adherence, which are critical factors in the success of fall prevention interventions.

The observed benefits align with prior research indicating that digital interventions can effectively reduce fall incidence and improve balance and mobility. For instance, a previous study demonstrated that e-counseling interventions, whether delivered via telehealth, video consultations, or mobile apps, address many of these barriers by improving accessibility and allowing older adults to engage in fall prevention strategies from the comfort of their homes [45]. However, earlier reviews often focused on in-person interventions, such as group exercise interventions, home visits, or physical therapy, to address fall risk in older adults [46]. Moreover, earlier reviews highlighted issues with adherence, often related to the logistical difficulties of attending in-person sessions consistently [18]. Previous reviews had not emphasized the role of digital tools as strongly, as the technological infrastructure and adoption among older adults were not as widespread [47]. With the increased availability and acceptance of technology, recent studies have shown that digital platforms can provide continuous monitoring and personalized interventions, a feature that was less prominent in earlier studies that focused on more generalized group interventions.

Additionally, previous reviews noted a lack of sustained follow-up, with many interventions being short term and failing to assess the long-term sustainability of fall prevention strategies [48]. Recent findings show that e-counseling, through regular follow-ups and remote monitoring, enhances adherence and promotes long-term engagement in fall prevention activities [49]. This continuous interaction is a marked improvement over older models, which often relied on intermittent or in-person check-ins. Another critical advancement in recent studies is the inclusion of cognitive training and the exploration of how e-counseling can improve not only physical outcomes such as balance and mobility but also cognitive function [50]. Earlier reviews primarily focused on physical outcomes, leaving cognitive aspects underexplored. Recent studies have demonstrated that e-counseling can provide a holistic approach by incorporating both physical and cognitive training into fall prevention strategies, offering a more comprehensive intervention [25]. In summary, compared to previous reviews, the recent findings reflect significant advancements in accessibility, adherence, and the integration of personalized care through digital tools, positioning e-counseling as a scalable and effective intervention for fall prevention among older adults.

Despite the promising findings from existing studies, several research gaps remain in the field of e-counseling for fall prevention among older adults. First, although prior studies demonstrate improvements in knowledge, confidence, and adherence to safety measures, evidence on long-term effects on fall rates and overall health outcomes remains limited. Second, most studies focus on specific populations or geographic regions, limiting generalizability to more diverse groups of older adults with varying health statuses and cultural contexts. Third, the impact of different e-counseling modalities (eg, video calls vs SMS text messaging) on engagement and effectiveness remains underexplored. Finally, although some studies report reductions in fall-related anxiety and increases in physical activity, further research is needed to determine how e-counseling can be integrated into broader health care systems and coordinated with in-person care to support comprehensive fall prevention strategies. Addressing these gaps may enhance the effectiveness and applicability of e-counseling interventions for older adults [24,51].

A multidisciplinary home-based telehealth intervention has also been shown to be feasible and effective in reducing fall risk among older adults, highlighting the importance of integrated and technology-supported approaches in fall prevention programs [52].

### Clinical Implications

The results underscore the potential of e-counseling as an integral component of fall prevention interventions in clinical practice. Health care providers should consider integrating e-counseling interventions into routine care for older adults, particularly those facing mobility challenges or residing in remote areas where access to in-person services is limited. By leveraging digital tools, clinicians can offer personalized care plans that adapt to each individual’s needs, enhancing adherence to recommended exercises and preventive measures. Moreover, the observed improvements in balance, mobility, and cognitive function suggest that e-counseling not only reduces fall incidence but also contributes to the overall well-being of older adults. Clinicians should be proactive in promoting e-counseling options as part of comprehensive fall prevention strategies, particularly in light of the growing acceptance of telehealth among older populations. Additionally, the role of regular follow-ups and continuous monitoring emphasizes the need for a structured approach to e-counseling. Health care professionals should prioritize ongoing engagement with patients through scheduled virtual check-ins, ensuring that individuals remain motivated and accountable in their fall prevention efforts. This ongoing support can foster greater confidence and satisfaction, leading to more effective long-term outcomes.

### Limitations

Several limitations must be acknowledged. First, the eligibility criteria restricted the inclusion of articles to those published in English and within the last 5 years (2019-2025), potentially excluding relevant studies in other languages or earlier research that could provide valuable insights into e-counseling for fall prevention among older adults. Additionally, the selection of articles was based on predefined criteria, which could introduce selection bias; focusing on specific databases may have led to the omission of studies published elsewhere or gray literature, affecting the review’s comprehensiveness. Furthermore, the included studies varied in their designs, methodologies, and outcome measures, complicating the synthesis of findings and limiting the ability to draw definitive conclusions regarding the effectiveness of e-counseling interventions.

### Conclusions

This scoping review shows that e-counseling represents a promising and scalable approach to fall prevention among older adults, particularly when interventions are personalized, culturally appropriate, and supported by ongoing monitoring. These findings can inform the development of integrated digital fall prevention strategies within health care systems and support policy initiatives aimed at promoting healthy aging. Future studies should prioritize long-term effectiveness, equitable access in low- and middle-income settings, and sustainable integration into routine care.

## Appendix

Multimedia Appendix 1 PRISMA-ScR checklist.

## Abbreviations

AI: artificial intelligence
MeSH: Medical Subject Headings
mHealth: mobile health
MMAT: Mixed Methods Appraisal Tool
PRISMA-ScR: Preferred Reporting Items for Systematic Reviews and Meta-Analyses Extension for Scoping Reviews
RCT: randomized controlled trial
VR: virtual reality
