# Supplementary material for: e-Counseling for Fall Prevention in Older Adults: Scoping Review
Source: JMIR Aging. 2026 May 26;9:e78444. doi: 10.2196/78444 (PMC13250495; doi:10.2196/78444)
Supplement: Multimedia Appendix 1 [file aging_v9i1e78444_app1.docx]

**PRISMA-ScR Checklist**

**Manuscript Title:** e-Counseling for Fall Prevention in Older Adults: Scoping Review
**Journal:** JMIR Aging

| **SECTION** | **PRISMA-ScR ITEM** | **REPORTED ON PAGE** |
| --- | --- | --- |
| Title | Identify the report as a scoping review | 1 |
| Abstract | Provide a structured summary including background, objectives, eligibility criteria, sources of evidence, charting methods, results, and conclusions | 1 |
| Introduction | Describe the rationale for the review | 1-2 |
| Objectives | Provide an explicit statement of the questions and objectives | 2 |
| Methods | Present protocol and registration information if available | Not applicable |
| Eligibility Criteria | Specify characteristics of sources of evidence used as eligibility criteria | 2 |
| Information Sources | Describe all information sources in the search | 2 |
| Search | Present the full electronic search strategy | 2 |
| Selection of Sources of Evidence | State the process for selecting evidence | 2-3 |
| Data Charting Process | Describe methods of charting data | 3 |
| Data Items | List and define all variables for which data were sought | 3 |
| Critical Appraisal | Provide rationale for conducting critical appraisal | 3 |
| Synthesis of Results | Describe methods of handling and summarizing data | 3 |
| Results | Give numbers of sources screened, assessed, and included | 3-4 |
| Selection of Sources of Evidence | Provide results of the search and selection process | 3-4 |
| Characteristics of Sources of Evidence | Present characteristics for which data were charted | 4-6 |
| Critical Appraisal Within Sources of Evidence | Present data on critical appraisal | 4-5 |
| Results of Individual Sources of Evidence | Present relevant data from included sources | 5-6 |
| Synthesis of Results | Summarize charting results related to objectives | 5-7 |
| Discussion | Summarize main results and evidence overview | 6-7 |
| Limitations | Discuss limitations of the scoping review process | 7 |
| Conclusions | Provide interpretation and implications of findings | 7 |
| Funding | Describe funding sources for included evidence and review | 7 |

**Notes**

- This checklist follows the PRISMA-ScR (Preferred Reporting Items for Systematic Reviews and Meta-Analyses Extension for Scoping Reviews) reporting guideline.
- The checklist should be uploaded to the JMIR submission system as:
  Multimedia Appendix 1.
